# Hygiene Publique

**Published:** 1837-04-01

**Authors:** 


### HYGIENE PUBLIQUE, PAR *A. J. B. Parent-Duchatelet*. 2 Tomes. Pp. 552 and 704. Bailliere, 1836.

M. DUCHATELET is already known to our readers, as the author of the remarkable work on Prostitution, which has been reviewed in the two last numbers of this Journal. The present one consists of a series of Memoirs on subjects connected with public hygiene, more especially in reference to the state of Paris and its environs.

A short biographical sketch of the author is prefixed by his friend, M. Leuret, to whom was confided the publication of the work. From this we learn that M. Duchatelet, renouncing the profession, in which he had fair hopes of a bright success, had devoted the last fifteen years of his life to the prosecution of a calling, which, although dangerous as well as repulsive and even disgusting, must be allowed to be of great importance, and one on which the health of large cities, and the comfort and convenience of individuals so much depend. He assures us himself, that he had traversed and examined almost every sewer in Paris, had conversed with almost all the workmen, had assisted them in their labours, had accompanied them to the most infected spots, had spent days and even weeks in the Augean stables of filth and nastiness in the neighbourhood of Paris—Montfauçon and Bondy—had visited repeatedly all the workshops of the tanners, the preparers of animal grease, glue, music strings, Prussian blue, &c. and that he thus acquired an intimate acquaintance with almost every hole and corner of the metropolis. He was long one of the most efficient members of the council of health (Conseil de Salubrité), and repeatedly applied to by Government for information relative to many of the public works. Numerous were the reports which he drew up and published at different times. The substance of these will be found in the two volumes under review. We propose now to give a short analysis of the most important of these. The first, to which we shall call the attention of our readers, is one on the past and present state of the dissecting-rooms in Paris.

An historical sketch of these, during the last century, is detailed with great minuteness. He mentions many curious particulars respecting the extreme difficulty of prosecuting anatomical studies in Paris, and the numerous obstacles which the teachers experienced in procuring bodies for dissection, during the early and middle parts of the last century. We were not aware, until we perused M. Duchatelet's work, that there had been no public or recognized amphitheatre of anatomy there, before the time of the great Dessault. To this distinguished surgeon was due, it appears, the merit of having overcome the national prejudices against the prosecution of dissecting, and of having first established openly rooms for the purpose. His example was followed, a few years afterwards, by several of his pupils, among whom we may enumerate Dubois, Lallemand, Bichat and Boyer. All these celebrated men were teachers of anatomy, and superintended for many years private dissecting rooms.

At this period, the supply of bodies must have been scanty and not easily obtained. Very few were procured from the hospitals; and hence recourse was necessarily had to the church-yards, which were then numerous within the walls of Paris. The state of the dissecting-rooms was, from all accounts, most shocking and repulsive. They were small, miserably inconvenient, being often situated in the attics of an inhabited house, filthy beyond description, and therefore not a little offensive to the neighbourhood. Complaints upon complaints had been made against the continuance of such a nuisance, and the local police of the town had repeatedly made attempts to correct and improve the state of things. But what with the state of agitation and confusion which prevailed for so many years, before Buonaparte seized the reins of government, and other causes which M. Duchatelet alludes to, it would seem that no effectual step towards any change was taken until the year 1806, when a commission was instituted to examine into the state of the medical schools, and more especially of the dissecting-rooms throughout Paris. The late Baron Dupuytren was one of the number, and to him was entrusted the charge of drawing up the report. Certainly none was better qualified to advise Government on such a subject. He himself had been for many years a teacher of anatomy in the metropolis, having been first the assistant, and then the successor of the immortal Bichat. The most important suggestion in the report was that, wherein the commission recommended that all the small private schools should be thenceforth abolished, and consolidated, as it were, into two great central establishments; one to be appertaining to the faculty of medicine, the other to be attached to the Hôpital La Pitié. We are told that Napoleon himself took considerable interest in these affairs, and that he had designed some important changes in many of the medical institutions of his metropolis. Certain it is, that about this time, 1806, he had given orders that the present decayed building of the Hôtel Dieu should be demolished, with the view of reconstructing it upon a grand and more commodious design, not only as a hospital, but also as a great central school of instruction.

This project, like many others of the same mighty architect, has, it is well known, never been carried into effect. Even the vile, filthy dissecting rooms, of which there used to be so many around the hospital, continued to exist for several years afterwards; and it was not until 1813 that M. Pasquier, then the Prefect of police, issued the ordinance, by which all the private schools were abolished, and the suggestion of the commission was carried into effect—viz. the establishment of only two great anatomical amphitheatres. For some time previously a very important change, in the mode of procuring subjects for dissection, had taken place. We have already mentioned that, in the time of Dessault, and of his immediate successors, the cemeteries within the barriers had yielded the supply of bodies for the anatomists; but, after Napoleon had ordained the removal of all the churchyards to the outside of the walls, and the disinterment of bodies was prohibited by severe penalties, the bodies of those who died in the hospitals and hospices of the metropolis were appropriated for the purpose.

The centralization of the dissecting-rooms gave great offence to many of the young teachers and professors of the private schools; and various attempts were made to evade the enactment. These attempts have gradually succeeded; and hence, at the present time, there are dissecting-rooms, not only at the La Pitié Hospital, and at the Ecole de Medecine, but also at the Salpetriere, Maternité, St. Louis, Beaujon, and Charité Hospitals. From M. Duchatelet's enquiries, it appears that, between twelve and fourteen hundred bodies are dissected annually at the La Pitié rooms, and not fewer than a thousand at those of the School of Medicine. Most of the English, and other foreigners, who go to Paris for anatomical instruction, dissect at the former.

Our author examines at great length the question, whether the existence of anatomical amphitheatres in a city is at all injurious to the public health; as, some years ago, it was often urged against these establishments, that they were so many "foci" of infectious emanation. He has quite satisfied himself that there is not a shadow of truth in this statement; and, among other arguments, he appeals with confidence to the healthy state of the Hôtel Dieu in former years, when this hospital was surrounded by numerous dissecting-rooms, some of which were even within its walls. He next adduces the testimony of a great number of eminent authorities to confirm his opinion. The first he quotes is the late M. Lallemand, who has left us an account of the dissecting-rooms first established by Dessault. They were, it appears, situated on the top story of an old decayed house. The number of bodies usually on the tables was from 50 to 60, and the number of pupils 200 or more. The rooms were very seldom cleaned, and even the debris of the bodies was not removed oftener than once a month. Nothing could exceed the abominable stench diffused over the immediate neighbourhood; and yet, says M. Lallemand, we never heard of any diseases, which might be fairly attributed to the presence of the dissecting-rooms, either among the students themselves, or among the inhabitants of the adjoining houses. Dessault himself used to say, that he really believed that the *odorous* air of his dissecting-rooms saved him from attacks of epidemic and other disorders, of which those hospital physicians and surgeons, who seldom or never dissected a dead body, seemed to be much more susceptible than himself. He took pleasure in repeating the old saying—"morte la bete, mort le venin;" and therefore he did not feel at all desirous, he said, to alter the state of the rooms under his charge.

M. Duchatelet next appeals to the testimony of Dubois, Dupuytren, Boyer, and of a host of others still alive, who have been, or still are, teachers of anatomy. They all agree in opinion, that it is quite an error to suppose, that the air of a neighbourhood is ever contaminated—so as to induce disease—by the emanations from dissecting-rooms, or that the students ever suffer from breathing the impure air of these places.

M. Duchatelet applied to M. Andral, among many others, for his opinion on this subject. The following is an extract from his letter:—"As to the diseases which medical students contract during their dissections, we have no good grounds for attributing them to cadaveric emanations. Gastro-enterites, meningites, and typhoid fevers, are very frequent among the students during the first year of their stay in Paris; but these diseases depend so little upon the mere circumstance of the 'sejour' of the students in the dissecting-rooms, that they are more common among those who have not begun to dissect. Indeed the mortality among the medical pupils is certainly not greater, than among an equal number of any other class of young men. The fatigues of study, the late hours, the intellectual exertion at home for the concours, &c. are much more hurtful to the constitution, than the labours of the dissecting-room. I have taken the trouble to ascertain the general health of the servants of the amphitheatres—and some of them pass day after day there without once going out—and it appears that they are quite as healthy in every respect, as other men."

With respect to the health of the students themselves, M. Duchatelet has taken great pains to discover, whether there are any good grounds for supposing, that it is affected by the air and the labour of the dissecting-room. According to his account, the most frequent complaint among these youths is dyspepsia, attended often with colicky pains and diarrhœa. But these symptoms are, says he, attributable much less to their engagements in the dissecting-rooms, than to the imperfect or improper diet, which the "res angusta domi" imposes upon many of them. Not a few of the French students, especially those who arrive from the provinces, are but scantily provided with even the absolute necessaries of life. M. Duchatelet assures us that he has known several, who have lived for weeks, aye and months too, upon stale bread, and an occasional glass of brandy.

It may be worth while to notice also the usual good health of the servants of the anatomical theatres. Many of them live within the premises; yet they do not suffer any direct inconvenience; and it has been often remarked that these men are singularly exempt from febrile diseases.

The mention of these "garçons" leads M. Duchatelet to allude to a practice, which seems to have been, some years ago, (and may be still), very common among them, but which probably is quite unknown to most of our readers. It appears that they were in the habit of collecting all the fat from the dissected bodies, and selling it to the enamellers, manufacturers of pearls, and other artisans, who require that the lamps, which they use at their work with the blow-pipe, should have a steady strong flame. Human fat, we are told, is better suited for this purpose, than that of other animals, from its remaining more uniformly fluid. Horse and dog grease is what is commonly used; and, as *such*, the fat from the dissecting-rooms had been sold. M. Duchatelet mentions that two thousand litres of human fat were found, some time ago, in the possession of one of the servants attached to an anatomical amphitheatre. This man confessed that he intended to have sold it, and that for several years he had made a good deal of money in this way.

The police applied to the Academy for information, on the means of distinguishing human fat from that of other animals. It seems from the following extract of the report, drawn up by Dupuytren at the time, that the question does not admit of an easy solution:—"Les graisses d'homme, de cheval, et d'âne ne peuvent etre distinguées entre elles, parcequ'elles ont toutes une coleur jaune, une concrescibilité tres faible, une très grande fetidité, et qu'elles se precipitent en globules."

But to return from this digression, to the question as to the influence of the exhalations from putrid animal bodies on the health of those engaged in dissection, M. Duchatelet mentions that, it has been conjectured by some, that the effluvia from human bodies are not so injurious as the emanations from those of the lower animals. It appears, however, that this idea is not warranted by experience.

M. Rousseau, who has, for six and thirty years, been the superintendent of the anatomical preparations at the Jardin des Plantes, assured M. Duchatelet, that neither he, nor any of his assistants, has ever suffered from their occupations, even during the Summer, although the bodies of the animals dissected there are often excessively putrid.

Our author, not satisfied with the preceding authorities, appeals to the testimony of Mr. Lawrence of London, contained in a letter which this eminent anatomist wrote many years ago, to Dr. Bancroft, and which is cited by Dr. Warren in the Boston Med. and Surg. Journal. Mr. Lawrence mentions the case of the servant, who, with his family, lived for ten years under the dissecting-rooms at St. Bartholomew's Hospital, without suffering the slightest derangement of health.

The means, which M. Duchatelet proposes for the purpose of purifying anatomical rooms, and to keep them in a *sweet* state, it is scarcely necessary to notice; as, in truth, they only consist in recommending cleanliness, free ventilation, the removal of all the debris as speedily as possible, and the use of the chlorides to the floors and tables.

He has described and delineated a dissecting table of a particular construction, which may be of use, when it is an object to preserve a body from putrefaction for a great length of time, or to examine one already much decayed. This table is boarded all round, and perforated with numerous holes, which communicate with a flue terminating in the chimney.

The effect of this contrivance, when there is a fire in the grate, is to cause a continued current of air to pass from within the room towards the chimney. The body on the table is thus exposed to a constant ventilation; and, moreover, the offensive effluvia are prevented from being diffused through the air.

From the subject of the dissecting-rooms, we pass on to the report of our author on the recent disinterment of a number of putrid bodies, from the vault of a church in Paris.

As soon as the uproar and bloodshed of the three "glorious days" had ceased, it became one of the earliest duties of the Provisional Government to provide interment for the unfortunate victims of the struggle.

In some of the streets the dead bodies lay in dozens, heaped and piled together; and already, in consequence of the hot weather, many of them had become most offensively putrid. There were upwards of two hundred deposited at the Morgue, and almost as many under the Pont Notre Dame.

At first it was proposed to dig a large grave for these in the Gardens of the Archbishop's Palace, which is situated close to the Cathedral; but the officers of the Hôtel Dieu very properly objected to this proposal, as the air of the hospital might have been thereby contaminated, and the health of the inmates seriously injured. The bodies, therefore, were put into barges, and floated down the river as far as the Bridge of Jena, opposite the Champs de Mars, and there buried.

M. Chevalier had the charge of superintending the removal; and, by the judicious arrangements which he made, not one of the workmen, employed in the offensive occupation, suffered. Large quantities of chloruret of lime, both in its dry and its liquid state, were used for the purpose of disinfection.

While these steps were taken at one part of Paris, the people in different quarters had, of their own accord, dug deep pits, wherever there was a vacant spot, and had buried the bodies in heaps. No injurious consequences ever followed, as far as we know, from this proceeding. But in one neighbourhood, that of the Rues Montmartre and Mont-orgueil, a number of bodies having been collected together under the portico of the church of St. Eustache, it was unfortunately resolved to deposit them in the vaults underneath. There they lay for some weeks, until the air of the church became so offensive, that no service could be performed in it, and the inhabitants of the adjoining houses began to complain of the fetid emanations. The subject was referred by Government to the Council of Health, for their advice as to what steps ought to be taken. At first it was proposed to do nothing more, than to re-open the vault, and to introduce a quantity of quick-lime over the spot where the bodies had been thrown in, for the purpose of accelerating their decomposition, and also of counteracting, it was hoped, the offensive effluvia. But this inefficient measure was very properly rejected. A committee, which contained among its members M. Labarraque, the famous chemist, M. Andral, and our author, was deputed to ascertain particulars, and to suggest what should be done. These gentlemen quickly determined on the necessity of removing the bodies, however decayed and putrid they might be. M. Labarraque proposed to superintend himself the operations. Having had all the windows of the church opened, he had a number of buckets, full of the solution of the chloruret, placed in different parts. The workmen also washed their hands and faces well with it. When an opening was made into the vault from the floor of the church, a quantity of the chloruret was thrown in, and one or two of the workmen (servants employed at the Morgue and in the public sewers) descended. M. Duchatelet immediately followed them.

He tells us, that the stench was not nearly so offensive as might have been expected. The floors and walls of the vault, and the dead bodies also, (some of which were excessively putrid,) were well washed with the chloruret. The bodies were then enveloped in separate pieces of coarse canvas which had been steeped in the solution, and were hoisted up into the church. The whole business was completed in little more than two hours, and not a single person engaged complained even of temporary inconvenience. The bodies were then conveyed to the great cemetery at Montmartre, and were all (43 in number), buried in the same grave.

The next memoir, which we propose to notice, is rather a lengthy and tedious one on the question, how far putrid effluvia affect the wholesomeness, and tendency to decomposition, of sound meat and other articles of food. The motive, which M. Duchatelet alleges for his minute examination of this subject, is that it has been repeatedly stated, even by professional and scientific men, that the health of the inhabitants, who live in the neighbourhood of slaughter-houses, dissecting-rooms, public sewers, &c., has suffered, not only from respiring a vitiated atmosphere, but also from their food becoming quickly corrupted.

To settle this question, he visited almost every hole and corner of Paris,—such as the neighbourhood of that most abominable sewer-like stream, the Riviere de Bievre near the Gobelins; many of the laystalls, and especially that incomparable stable of filth and nastiness, the enormous slaughter-house at Montfauçon, where not only between 12 and 13,000 horses are killed yearly, but almost all the ordure of Paris is collected together; the abodes of the workmen employed in the disgusting occupation of making music strings from the intestines of animals; not to mention the houses of the servants engaged in the Amphitheatres of Anatomy. The result of all his enquiries has been, that the almost constant effluvia of these places has very little or no effect in corrupting, or otherwise affecting, the wholesomeness of any article of food. Meat, broth, and vegetables, seem to be as easily preserved there, as in the healthiest and most airy districts of the metropolis.

Not content with this mode of enquiry, our indefatigable author carried on, for a period of five months, a series of direct experiments, in his own laboratory, to determine the question under consideration. He kept different articles of food, such as blood, milk, chicken-broth, mutton-broth, beer, wine, gruel, solutions of starch and gum, as well as meats of various sorts, surrounded with vessels filled with the most putrid and putrefying substances; and he found that, although they all (but in different degrees), acquired an offensive odour, they speedily lost this on being exposed to the air; and that they did not become decomposed sooner than other portions of the same substances, which had been kept in a pure place. It may be useful to know that, in conducting these experiments, M. Duchatelet discovered that the substances might be preserved even from acquiring the slightest offensive odour, by covering their surface with a thin layer of oil; and also, that boiling will effectually remove it, after it has been acquired. The thicker and more viscid a fluid is, the less readily does it become impregnated with the fetid effluvia. Hence pure water is most quickly tainted; whereas solutions of gum, starch, gelatine, &c. are so, very slowly. We have, however, already stated that this taint may always be removed by exposing the fluid afterwards to the free air, and especially by boiling. It thus appears that putrid emanations act merely as any other odoriferous substance: the effluvia penetrate more or less easily the substance which may be exposed to their influence; but they do not cause any permanent or molecular changes in it, nor do they seem to accelerate, in the slightest degree, the tendency to decomposition.

M. Duchatelet has suggested a simple and very ingenious contrivance for the preservation of meat, and of other corruptible substances. He finds fault with the common plan of merely suspending them from the ceiling, as if the air was cooler there than nearer the floor. If, however, a tray holding pieces of ice, or a vessel of iced water be hung over them, a current of air from above downwards, and vice versa, will be occasioned: the cooled air descending, and being replaced by a volume from below. He also suggests the use of a table, constructed on the same principle as the dissecting table, which we have already described. These hints may possibly be useful. M. Duchatelet mentions, that one of the famous shops for "comestibles," in the Palais Royal, is provided with a table, such as he recommends.

Our attention is now called to another memoir, which although long—extending to 130 pages—is, nevertheless, very interesting. It is occupied with a minute account of those disgusting, but very necessary, establishments for the slaughter of old decayed horses, and for the flaying of all the dead dogs, and cats, not only in Paris itself, but also such as are brought from several miles round. These establishments are called "chantiers d'equarrissage;"* and, from what our author says of them, it would seem that the filth, stench, and nastiness of them is most insufferably horrible. Addressing his countrymen, he says: "You may well be proud of your markets and of your abattoirs, of your fountains which wash and refresh your streets, and of your sewers which dry and cleanse them; but is it not too bad, that every time you go out, and come into your city, 'qui se pretend la capitale du monde civilisé' (!), you run the risk of being suf-

* They are known, we believe, in this country under the name of "Knackers."

focated by the vile emanations from the slaughter-houses and heaps of ordure, 'dont l'aspect seul fait reculer d'horreur ?' "

The largest of the "chantiers d'equarrissages" is situated at Montfauçon, a mile or so from the north-east extremity of Paris.* There is another, of smaller extent, on the opposite side of the river, not far from the Hospice de la Salpetrière. Various expedients have been proposed, not only by private speculators, but also by the public authorities, to obviate and counteract, if possible, the horrible loathsomeness of these establishments. At one time it was ordered, that the carcases of the beasts should be stripped at once, and that all the flesh, fat, and garbage should be daily buried in deep pits. But the proprietors, many of whom have made immense fortunes, petitioned against this most oppressive (at least to them) regulation. They had been in the habit not only of melting all the fat, which fetched a high price, but also of selling the flesh, bones, &c. to the farmers, for the purposes of manure; not to mention the profitable sale of the garbage, hoofs, &c.

In 1812, three gentlemen, who had a high name as most able manufacturing chemists, suggested an ingenious plan for diminishing the offensiveness of the "chantiers." It consisted in hanging up all the stripped flesh &c. in leaden chambers, and there exposing it to an atmosphere of steam for several hours. The fatty matters, being melted by the heat, drop down, and collect into a stream; while the fleshy parts are sodden, and therefore not liable to putrefaction. They are then to be pressed by a machine into large pieces, which are ready for sale to the sellers of dog and cat meat, to the farmers, and to the manufacturers of Prussian blue, &c. The Emperor Napoleon granted letters patent to these gentlemen; but we are not informed whether they have ever carried their project into effect.

In 1822, M. Dupuy, the professor in the Veterinary College at Alfort, petitioned Government to be appointed Inspector of the various slaughter-houses in and about Paris (to which, he states, there are annually brought between 7 and 8,000 horses—12,000 dogs, and 6,000 cats†), for the purpose of prosecuting his researches in comparative pathology; but this request was not complied with.

One of the abominable evils resulting from the "chantiers d'equarrissage" around Paris is, that the flesh of the dead horses has been secretly sold as food to the inhabitants, on many occasions. During the first revolution, it is well known, that there was, for a considerable length of time, a great scarcity of provisions; and we have the authority of M. Huzard, who had ample opportunities of ascertaining the truth, that a large portion

* Several years ago, a company of "equarrisseurs" was set going in another district of Paris, and for a considerable time their affairs appeared to be very thriving. Unfortunately, however, for the concern, it was discovered that the pork and sausagemongers were in the habit of keeping an immense number of grunters near the "chantier," for the purpose of feeding them with the vile garbage of horse, dog, and cat-flesh. For several months, Paris might have been taken for a metropolis of Jews or genuine Musselmen; not a sausage or piece of pork were to be seen any where!

† This statement of the number of beasts taken to the "chantiers" differs, it will be noticed, from that given by our author. We are inclined to believe that M. Duchatelet may be depended upon for the accuracy of all his assertions.

of the animal food, consumed during that period in Paris, consisted of horse-flesh. In 1803, the surreptitious sale of this was again discovered; and repeatedly in 1811, when, it is well known, all articles of food were exceedingly expensive, and there was much want and distress among the lower orders. The commissioners of police seized large quantities in the houses of several of the petty victuallers, in the indigent quarters of the metropolis. M. Pasquier, at that time head magistrate of Paris (Prefect of police), applied to the council of health for their opinion, whether the use of horse-flesh, as food, is injurious to health, or not. Like many other commissions, the one, appointed to report, returned a very dubious answer. M. Pasquier, therefore, acted upon his own judgment, and positively interdicted the sale of the flesh from the "chantiers d'equarrisage" *upon any account* or *for any purpose.* The very strictness of this injunction unfortunately frustrated its intentions. The vast number of dogs in and around Paris, not to mention the carnivorous animals in the Jardin des Plantes could not, it was alleged, be fed in any other way, but by the meat obtained from the horse-slaughter-houses; and hence permission was afterwards granted for the sale of this meat to certain persons, but only for the purpose now mentioned.

It seems, however, that as recently as 1817, there was a quantity of horse-flesh sold for human food in Paris; and in 1825, the committee, appointed to inquire into the state of the various laystalls about Paris, actually proposed to Government to permit the sale of horse-flesh for human food under certain regulations. This strange suggestion was very properly rejected.* It was, however, again proposed by some, after the last revolution in 1830 when great numbers of the poorer classes were quite destitute and starving. But although the practice was prohibited, it would seem that, even in the present day, a large quantity of horse-meat is sold in Paris. The following are the words of one of the commissioners of police, published not long ago:

* Our author, indeed, seems rather inclined to permit the sale of horse-flesh, under certain restrictions. He tells us, that many of the men employed in the great slaughter-house at Montfauçon make use of it constantly, and that they do not suffer any bad effects from the practice. In Denmark, and also, we believe, in Sweden, the public sale of horse-flesh is authorised; and Baron Larrey informs us, in his great work on Military Surgery, that the wounded of the French army, in the Egyptian and Russian campaigns, were fed for weeks and even months upon horse-flesh, without any injury to their health. "The flesh of the horse (says he) makes a very good soup, especially if some lard is added to it; or it may be broiled, or prepared as 'bœuf à la mode,' mixed with proper seasoning. The liver, too, was highly esteemed by our soldiers in Russia. During the siege of Alexandria, the health of the troops was mainly preserved by the regular supply of horse-meat to them. It contributed very materially to the stoppage of a scorbutic epidemic, which had appeared among them. I repeatedly partook of the meat myself, and found it both palatable and nutritious. After the battles of Eylau and of Eslingen, the wounded were fed upon horse-flesh for several days; and when, at length, oxen were procured, the soldiers did not recognize the difference. The cuirasses of the cavalry regiments served as utensils to boil the flesh in, and gunpowder was used in the place of salt, of which there was a complete want in the camp. 'Ce bouillon fut d'une tres bonne qualité.' Marshal Massena partook of the same fare, and highly approved of it."

"Il est de notorieté publique que l'on vend, à raison de quatre sous la livre, chez divers restaurateurs de la capitale, de la viande choisie de cheval, nouvellement abattue; que ceux, qui ne peuvent ostensiblement faire entrer dans le jour de cette viande, vont à une heure, et à un lieu convenu, en jeter, par dessus les murs, des morçeaux considerables, qui sont à l'instant ramassés..... Ceci se pratique tous les jours."

It would seem, from what our author says, that even dogs and cats are often sold in Paris for human food. His own words are: "We have never visited this establishment (the slaughter-house at Montfauçon, appropriated to the skinning of these animals), without finding a number of them 'ouverts, depouillés, et troussés avec soin, tout prets enfin à faire cuire pour le repas auquel ils devaient servir." !!

But to return to the more immediate subject of our inquiry, that of the "chantiers d'equarrissage," we must refer such of our readers, as may wish to know particulars concerning them, to M. Duchatelet's work. There is a minute description given of the immense establishment at Montfauçon, in the immediate vicinity of Paris. He estimates the number of horses taken there, either dead or to be killed, at between 12 and 13,000 annually. A considerable proportion is sent from the country, within several miles around Paris The account which he gives of the wretched famished animals led there, in dozens, to be slaughtered, furnishes a sad commentary on the mercenary unfeelingness of man! The number is always greatest towards the end of the year; because *then* a number of the poor animals, which had been regularly *worked out* during the previous Summer and Autumn, are not deemed by their masters worth the expense of their *keep* through the Winter. They are therefore condemned to the slaughter-house, where they usually fetch from 10 to 20 francs a head.

There are four different methods in which the animals are killed: by blowing air into an opened vein, by pithing, by bleeding, or, lastly, by felling on the head. The third of these methods is the one usually adopted. A long knife is introduced into the thorax, in the direction of the arch of the aorta which is almost always divided: the animal staggers for a minute or so, then drops down, and expires in convulsions.

The only inconvenience of this method is, that the animal sometimes does not fall down on the spot, where he stood when wounded. Hence, when there are a number of horses crowded together in a small space, the "equarrisseurs" very frequently employ the last-mentioned method, felling; as the animal usually falls down on the spot. The animal is quickly stripped of its hide; the flesh is next cut off from all the bones, and heaped together for sale; the viscera are thrown together in large masses, and the carcase is then carried off to a corner in the yard.

We have omitted to state that the hair of the mane and tail is always cut off, before the animal is killed; but this, usually, has been done before the beast is taken to the slaughter-house. The hides are sent off to the tanners (most of whose establishments are on the other side of the Seine, adjoining the filthy stream of the Bievre, near the Gobelins); the flesh is divided into different heaps, some for human food (!), others for dog, pig, and cat-meat,*

* We are also told that many of the poultry-feeders about Paris give horse-meat to their ducks, geese, and fowls.

and for the supply of the carnivorous animals in the Museum of Natural History. Some of the large dogs, kept in and about Paris, are trained to go by themselves to the slaughter-house, for a weekly supply at a time. The men know them, and fasten a large piece of the flesh round their necks, and off they go, thus loaded, to their masters' houses. What is not disposed of in these manners is, along with the garbage of the viscera, sold for manure, or to the manufacturers of Prussian blue, &c. The small intestines are sometimes used for the manufacture of the large coarse gut-strings. With respect to the blood of the slaughtered horses, M. Duchatelet expresses his surprise that it is not turned to more profitable account. Often it is allowed to flow on the ground, into which it soaks, forming a compost of most disgusting fetor; this is afterwards mixed with the dung, and sold as manure. If the blood was collected and separated, by stirring, into its liquid and its solid part, the former might be advantageously sold to the refiners of sugar, &c. and the latter, when dried, might be used for the food of animals, and even of man (!), or to some manufacturers. Most poultry will eat blood readily. A curious phenomena has been observed among the hens in some of the "chantiers:" the eggs which they lay have no shells. At present, the chief consumption of the fibrine of the blood (dried) is in the way of exportation, to the colonies, for manure. The tendons and hoofs are used for the preparation of glue, and in the manufacture of combs, &c. The horse-shoes are sold either as old iron, or to be again used as shoes; and the nails are collected together and sent into the provinces, where they are generally used "pour garnir les sabots" of the peasantry. The fat is collected into heaps, melted, and sold at a high price to the enamellers, manufacturers of pearls, and in general to all those who use the blowpipe in their work. The flame of a lamp, fed with horse-oil, gives a more steady flame, than with almost any other. This oil is also used in the manufacture of soap, for softening harness leather, and sometimes in the preparation of gas.

We have thus disposed of almost all the soft parts of the animal; and the reader may now enquire, what becomes of the bones. At one time they were used, in great quantities, in the *building of the walls (!)* in and around Paris, especially of such as surround the marshes and gardens. The bones were mixed up with moistened earth, which on drying was held together effectually. These walls were very common in the Faubourg Saint Marceau, and in the neighbourhood of the Temple, and the Barrier des Tourneaux—places near which the "chantiers d'equarrissage" have been usually situated. Many of the walls have of late years been destroyed, for the sake of the bones; but not a few are still standing.

The broad flat bones are used for coarse cutlery, and in the manufacture of toys; but the chief consumption of horse skeletons is by the manufacturers of ammonia, of animal charcoal, &c. When deprived of their gelatine, the bones are ground down and then sold for manure to the land.*

Having thus discussed the economical uses of the "chantiers," our au-

---

* Our author states, that in no district is there so large a consumption of bones for this purpose, as in Yorkshire. The quantity of bones sent to that county from London is quite enormous; amounting annually, it is said, to 33 millions of kilogrammes (a kilogramme is rather more than two pounds of avoirdupois weight).

thor proceeds to examine into the health of the workmen, engaged in the disgusting occupations of these places. With his accustomed zeal he has accumulated a great number of facts, to prove that the men are quite as healthy, or even more so, than most others of the lower classes.

We are told of the "santé la plus florissante" of the men, the "santé brillante" and the "fecondité remarquable" of many of the women, and the "force et mine admirables" of the infants, who are sometimes cradled "dans l'interieur d'une carcasse, comme dans un berçeau!" The same holds true of the manufacturers of gut-strings, and of the tanners, and of their families: they are in general remarkably healthy. What is especially worthy of notice, is, that the men very seldom suffer any inconvenience from wounding themselves, while engaged in their operations. The wounds, it seems, heal quickly; and it is rare that any case of carbuncle or of the "pustula maligna" occurs. "We shall mention," says M. D. "only one other circumstance in reference to the health of the workmen in the 'chantiers;' and that is, the remarkable exemption from illness, which they enjoyed, during the destructive prevalence of the cholera in Paris. There were far fewer patients admitted into the hôpital St. Louis, which is situated in the district of the 'chantiers,' than into any other hospital in the metropolis."

We do not wish it to be supposed, that, by the statements which we have now been making, we approve of the abominable nuisance of these establishments; but still it is a duty, which we owe to the public, to conceal no well-ascertained facts; and certainly one of these is, that the cholera affected scarcely any of the workmen now alluded to.

But we must now leave the "chantiers d'equarrissage," and pass on to another subject, which our readers may think to be still more uninviting— the state of the "fosses d'aisance" in Paris. M. Duchatelet, however, animated by a most laudable zeal to improve the condition of every class of society in that gay city, devoted much of his time and of his attention to this subject. "He had not," says his biographer, "the repugnance which these places naturally excite; I had almost said that he liked them (je dirois presque qu'il les aimait)! An anecdote told of him gives proof of this. Being present at a gay fete in the Hôtel de Ville, he could not help saying to one of his friends, 'J'aime centfois mieux aller dans un egout que de venir à cette reunion: on ne me verra plus ici!' Under such guidance, we shall not hesitate to accompany him in his rambles.

The state of the "fosses d'aisance" in Paris is certainly one of the greatest reproaches to the metropolis, which is so boastfully called by its inhabitants "the centre and mart of civilisation and refinement."

Many of our readers are perhaps not aware that, with the exception of a few insulated public buildings, such as the Palace of the Tuileries, the Hôtel des Invalids, the Bicetre Hospital, and two or three others, no dwelling in Paris is provided with a water-closet on the English construction: (latrine à l'Anglaise.) The reason of this great defect is no doubt, because the public sewers of the place are not numerous, and most of them are very imperfectly constructed. There are only a few large ones, crossing the metropolis in different directions to carry off the water and filth in the kennels of the streets, and the fluid refuse of manufactories; but the houses themselves have no communication with these sewers by means of private drains. Hence the only means, of getting rid of all the accumulated foul water, is to throw it either into the street, (to be carried off by the kennel there,) or into the privies, there to collect, until they are cleared and cleaned out—which operation is usually done once or twice a year in the better classes of houses, and only once every two or three years in the filthier parts of the metropolis.

Those, who have been accustomed to reside in London, and in the larger provincial towns of England, are indeed surprised, and not a little offended, by the state of things in Paris. The "fosses d'aisance" of a private house or hotel are frequently disgusting beyond description to more senses than one; and the practice of resorting to those of a more public description happily does not well suit English notions of decorum. Every thing is so admirably *appointed* with us at home, that it is only when we leave this highly favored, (however heavily taxed,) country, that we are sensible of the many conveniences and comforts, which we are almost unconsciously enjoying.

Our author informs us, that the expense of emptying and cleaning the privies in Paris has of late years greatly increased, in consequence of the improvements which have been made in their original construction. Formerly they were nothing better than holes or pits, dug in the ground, and walled on the sides. The urine, therefore, and all other fluid refuse which was thrown down, gradually soaked into the soil, and the worthy people of Paris did not trouble themselves where it went, or what became of it. But now, and for some time past, the "fosses" are built all round, being paved on the floor, as well as walled on their sides. The *advantage* (?) of this improvement is, that there is a vast accumulation of fluid as well as of solid filth collected during several months, until the period of emptying and cleaning comes round. The horrible offensiveness of this operation we need not describe. The filth is carted away to the great metropolitan laystalls at Montfauçon and Bondy, and there piled up in vast heaps, to undergo certain changes of fermentation and desiccation, before it is sold as manure to the farmers, or until it is exported to the provinces or to the French West India islands.

All this appears to the English reader most incomprehensibly disgusting. But, "what," says our author, "is Paris to do with this enormous mass of ordure? The construction of our houses does not admit of its being carried away by the sewers; and, moreover, not only should our agriculture suffer from the want of a most profitable manure, if this were done, but the Parisians would be shocked also, were they to suppose that the waters of the Seine, (from which, until very recently, all the supply of the metropolis is derived,) were contaminated with the contents of the fosses."

Various expedients have been proposed at different times to obviate, as far as possible, the stench and loathsomeness of the closets. The nature of almost all these experiments has been, to endeavour first to separate the urine and other fluids from the solid contents, and then to remove the former either by pumping it off, or by withdrawing it in some other manner.

By far, however, the best method is that proposed by M. Cazenave in 1818, and which consists in the adoption of moveable closets. Strange to say, this method has never met with encouragement from the municipal authorities, although the advantages, not only in respect of superior cleanliness, but also of diminished expence, are very obvious. M. Duchatelet very properly says, that no privy, however well and compactly constructed, can prevent the oozing of the water through its floor and walls. If such be the case, there must necessarily be a permanent focus of foul exhalation in every dwelling; and when, says he, we consider the numerous other agents all tending to render the abodes of the poor unhealthy, can we be surprised that low putrid fevers are so prevalent in Paris? Besides this consideration, there are others which deserve to be noticed. It is well known that there are numerous wells in different parts of Paris, and that many of the inhabitants derive all their supply of water from these. Now not a few of them are situated in those very neighbourhoods, where the "fosses" are the worst constructed, and the least attended to. The urine, therefore, permeating the soil, must necessarily contaminate the springs, from which the wells are fed. If there was occasion for further argument, we might allude to the melancholy circumstance, that scarcely a season passes over, without some accident to the scavengers employed in the disgusting duties of their calling. If we take up the Annales d'Hygiene, almost every number contains some lamentable instance, or another, either of death from asphyxia to these men, or of the horrid crime of infanticide, where the child has been concealed in the privy. These accidents could not occur, if the system of moveable "fosses" was generally adopted.

But it is especially in reference to the benefit which agriculture might receive, that our author strongly recommends the adoption of these contrivances. "Not only," says he, "would none of the solid matter be lost, but the mass of it would be very notably increased. Putrefaction and fermentation cause a very considerable portion to be dissipated; and moreover we are quite certain that it is less rich as a manure in its stale, than in its recent state."

It will naturally occur to our readers to enquire, what does M. Duchatelet recommend to be done with the fluid contents of the "fosses." The common practice hitherto has been, to cart it away along with the fæces, in immense casks, to the laystalls at Montfauçon and Bondy—there to be employed in the manufactory of ammonia, &c. and the refuse to be afterwards carried off by the large sewers into the Seine. Before detailing his own proposal, our author enters upon a minute and circumstantial enquiry, whether the waters of the Seine would be contaminated, if all the urine of the metropolis was discharged directly into its channel. Objections have often been made, even by able men, to this plan; and the French government has allowed itself to be influenced by these objections, in many of their municipal regulations. But it appears to have been forgotten all the time, that the sewers disgorge their contents into the river, and that many of these, as that of Montfauçon and Amelot, communicate with it *above* Paris. Where is the difference, in point of salubrity, between the conveying of the urine and other offensive fluids directly into the Seine, and the removing of it first to one large depositary, before it is carried off into the river by the sewer in the neighbourhood? M. Duchatelet, however, not satisfied with giving this most satisfactory reply, proceeds to show that the dread of contaminating the waters of the Seine is utterly groundless. He appeals to an admirable report of M. M. Fourcroy and Hallé, who have clearly proved, that the relative proportion of the fluids discharged daily into the river, with the actual amount of its mass, is so small and inconsiderable, that they can have no appreciable effect in vitiating it for almost any purposes, domestic or economical.

He calculates that the annual quantity of matters from the " fosses d'aisance" in the metropolis amounts to about 103,000 cube metres. One fourth of this may be supposed to consist of fæculent contents; and the remaining three fourths, or 77,100 cube metres, to be fluid. We may therefore estimate the daily quantity at 211 cube metres.

Now the average volume of the water of the Seine, even in a dry season, may be taken at 30,710 times larger than this quantity—211 cube metres. During the Winter and Spring months, and after any continued fall of rain, the preponderance is vastly more considerable. If we take the medium quantity of water in the Seine, as it passes through Paris, the flow may be estimated at 255 metres in the second;—a quantity, which exceeds that discharged into it from the sewers by at least a million times.

The conclusion from all these calculations is, that the fluid contents of the " fosses" might be discharged directly into the Seine, without any danger of contaminating its waters.

Having resolved this point, M. Duchatelet proceeds to enquire, what would be the most convenient and appropriate method of conveying these fluid contents to the river.

Our readers already know that this cannot be effected very easily in Paris by means of the sewers, in consequence of the want of private drains communicating with them. It is to be hoped that the Parisians will at length be convinced of the utility, or, we should rather say, the absolute necessity of these; and that the present method of carting away all the fluid as well as the solid contents of the " fosses" will be gradually abolished. Many a year however must pass over before this can be done; and until that fortunate time arrives, M. Duchatelet suggests that, if the moveable " fosses" were universally adopted, and emptied daily of their fluid contents, these might be advantageously got rid of, by discharging them into the kennels in the middle of the streets—at least in those places of the metropolis, where there is a sufficient supply of pure water to keep up a constant current along these kennels.

This proposal may, on first consideration, appear very objectionable; but our indefatigable author assures his readers, that he has quite satisfied himself that the addition to urine of from four to six times its quantity of water will deprive it of all its offensive smell. Since the opening of the Canal d'Ourcq (one of the great works of Paris recently completed, and one, by means of which every part may be abundantly supplied with water), there can be no scarcity of water; and the proposal therefore of M. Duchatelet, with the view of inducing every householder to become a subscriber to this great undertaking, is " qu'il sera dorenavant permis aux particuliers d'envoyer sur la voie publique les urines et les produits liquides des fosses d'aisance; mais seulement lorsque l'eau de l'Ourcq arrivera chez eux, et lorsque son ecoulement pourra se faire, sur cette voie publique, en assez grande abondance pour que ces matieres soient sans odeur fetide."

Before quitting the subject of the " fosses d'aisance" M. Duchatelet offers a few remarks, as to the best method of employing the solid contents. For the purpose of destroying their effluvia and of preventing their fermentation, he suggests that a quantity of animal charcoal, or, in lieu of this, which of late years has become very valuable and therefore very expensive, of calcined peat, or of the refuse of tan yards, &c. be mixed with the fæculent matters, before and after their removal from houses.

By the adoption of such simple means, much of the inconvenience and offensiveness of the present processes might be avoided; and the heaps of ordure accumulated in the laystalls, which in the present day are so disgusting, would be comparatively much less putrescent, and therefore better fitted for transportation as manure, either to the provinces, or to the colonies abroad.*

"In conclusion," says our author, "the changes now proposed in the construction and regulation of the "fosses d'aisance" are of such high importance, and the results, which might be obtained from their adoption, are so useful and so extensive, that they would suffice to distinguish and to recommend to the gratitude of future ages the names of those administrators, who may happily enforce them. This glory is reserved for the present two Prefects of the department of the Seine, and for the Municipal Council of Paris!!"

So much for the "fosses d'aisance of Paris. But before quitting the subject, it may be proper to allude to M. Duchatelet's 14th Memoir, which is entitled "Researches to Discover the Cause and the Nature of the Alarming Accidents, which occurred at Sea on board a Vessel loaded with *poudrette*." The vessel it seems sailed from Havre with a cargo of this most disgusting manure, and was bound for Guadaloupe. In the course of two or three weeks after leaving port, a scorbutic fever appeared among the crew. One half of them died, and the remainder, when they reached their destination, were miserably ill. Our author was appointed by the Marine to investigate into the particulars of the case, when the vessel returned to France.

It is not our intention to follow him through the details of the report, which he drew up on the occasion. Suffice it to say, that he came to the conclusion that the *poudrette* had been shipped, when it was too wet; that it ought to have been better dried; and he very properly suggests that a quantity of lime should be mixed with it, before it is taken on board any vessel. The effect of the lime is to absorb moisture and to counteract putrefaction and fermentation: besides it is well known to be a useful manure itself.

If we (the Rev.) were to give an opinion on the subject, we should say that no vessel should be allowed to carry such a putrid cargo—certainly not when bound to a tropical climate. Is it not strange that the fertile and luxuriant soil of the West Indies should require dung, as manure, from Europe?

The sixth memoir on the sewers of Paris, is the most elaborate one of the collection. The author very justly remarks, that the position of the French metropolis, lying as it does in a nearly horizontal valley, renders the necessity of a proper system of drainage the more imperative, while it adds greatly to the difficulty of effecting this most important object.

* This sort of manure, to which the French give the name of "poudrette," seems to be an article of very considerable commercial importance in France.

Much, he admits, has been done; that much remains to be accomplished, before Paris can be called a clean, or well appointed city. If there were a few more such zealous labourers as our author, the good work would be quickly advanced.

Multitudes are hourly traversing the streets of the gay metropolis, engaged in their business or their pleasure, without for one moment considering, or perhaps without being at all aware of, the fact, that they are passing over subterranean and therefore unseen, but most important and necessary structures, without which the existence of the health of the inhabitants would be speedily compromised, and the streets rendered almost impassable. They do not remember, that a number of their fellow citizens voluntarily condemn themselves to pass their lives in subterranean canals, exposed to all sorts of loathsome offensiveness, and doomed not unfrequently to perish while engaged in their revolting occupations. And yet, without the assistance of these men, a great part of Paris would become quite uninhabitable, and must necessarily relapse into the state of an unhealthy marsh; such as it was three or four centuries ago. It is well known that the site of the Louvre, of the Tuileries and its gardens, and of the Champs Elysées, was formerly a swampy morass. The ground is very low, and used to be generally overflowed by the river during the Winter.

M. Duchatelet commences his memoir by alluding to the cloacæ of ancient Rome, which are always viewed among the mightiest monuments of its greatness. Not satisfied with committing the superintendance of these important structures to the care of the first men of the state, the people of Rome invoked the protection and assistance of certain divinities to preserve them. They had their god, Sterquilinus, and their goddesses, Cloacina and Mephitina, under whose special patronage all the public cloacæ were believed to be.

Such was the consequence attached by the Romans to those great works. M. Duchatelet gives a very good and instructive account of them; and then proceeds to examine the state of the sewers in his own metropolis.—Certainly no one was better qualified than he to reprove and to instruct his countrymen on this subject. He had devoted, as we have already said, several years of his active life to the investigation of it. He had not only traversed every considerable sewer in Paris, and had conversed with all who knew any thing of them, from the highest member of the Academy, to the lowest of the workmen, but he had even repeatedly assisted the latter in their occupations.

"J'ai surmonté sans hesiter la repugnance et les dangers inseparables de pareilles recherches; j'y ai sacrifié mon temps, et mon argent, et ma peine; je ne puis dire les demarches que j'ai faites, les fatigues que j'ai eprouveés, et les contrarietés de toute espece qu'il m'a fallu endurer." Such was the discipline which our excellent author submitted to.

He first gives an historical sketch of the sewerage of Paris, from the earliest period down to the present time. The perusal of this sketch will well repay the readers, who may take any interest in the past and present state of the French metropolis, and more especially those who at the same time are fond of geological researches, respecting what has been called, by Cuvier and Brogniard, the basin of Paris; but it is impossible for us to do more than merely to allude to it.

In another part of this article, we have explained in what manner the contents of the "fosses d'aisance," are got rid of. They are not discharged into the sewers, but are conveyed away to the great depositaries at Montfauçon and Bondy.

In this respect, the sewers of Paris are greatly inferior in point of utility, as well as of cleanliness, and general management, to those of ancient Rome, and of London and other British towns. Not only are their dimensions much smaller, but their course also is uneven and irregular, and hence the current through them is slow and apt to stagnate.

It is to these causes that we are to attribute the numerous accidents, which occur every year to the workmen employed in cleansing the sewers of Paris. Such casualties are never heard of in London; whereas in Paris they are very frequent.

The Parisian sewers, every now and then, become obstructed from the deposit and accumulation of their solid contents; a large quantity of carburetted and sulphuretted hydrogen gasses is evolved, and the workmen, who are exposed to the effluvia, are apt to be asphyxiated.

Another topic, which has given rise to repeated discussion among men of science, as well as among the municipal authorities of Paris, is the question, how far the waters of the Seine, from which almost the whole supply of the metropolis is derived, are apt to be contaminated by the discharged contents of the sewers.

Numerous experiments have been performed to ascertain, whether there is any appreciable difference in the water of the river above the city (say at the Bridge of Austerlitz), from that lower down (at the Bridge of Jena). The result of all these experiments has been, to shew that chemical analysis does not discover any difference. Hence it has been concluded, that the contents of the various sewers, as well as of the filthy streams, such as the Bievre, which flow into the Seine, are quite neutralized by the comparatively enormous mass of water in the river.

This may be the case, says our author, in Winter, and after heavy falls of rain; but during the Summer months, especially when the season has been very dry, he has himself repeatedly observed, that the water of the river has been foul and discoloured, even as low down as Sevres, from the foreign matters discharged into its channel where it flows through Paris. The left or South side of the river is always infected in a greater degree, and for a greater length of time, than the opposite side—owing to the contaminating stream of the Bievre, on the banks of which most of the offensive manufactories are situated. Perhaps, says he, at no part of its course, is the Seine so muddy and filthy, as where it passes under the Hôtel Dieu Hospital.

Here we must abruptly stop. Our readers, no doubt, would like a little more of M. Duchatelet's company. He has infected both them and ourselves with a portion of his own enthusiasm, and, for our own parts, we have almost doffed our critical dressing-gown, and, tucking up our sleeves and breeches, prepared to descend into the sewers and the *fosses* with a true hygienic fervor. But, alas! a mandate most imperious arrests us. That literary autocrat, the printer's devil, tells us that there is room for no more "copy." What can we do? Our only resource is to come to a full stop, and introduce what observations remain in another portion of the Journal.*

* See Foreign Periscope.

